# Comment on ‘Diagnosis of Sarcopenia by Evaluating Skeletal Muscle Mass by Adjusted Bioimpedance Analysis Validated With Dual‐Energy X‐Ray Absorptiometry’ by Cheng et al.

**DOI:** 10.1002/jcsm.13587

**Published:** 2024-11-07

**Authors:** Hyunjee Kim

Dear Editor,

I am writing to express concerns and seek clarification regarding a paper published in the *Journal of Cachexia, Sarcopenia and Muscle* titled ‘Diagnosis of sarcopenia by evaluating skeletal muscle mass by adjusted bioimpedance analysis validated with dual‐energy X‐ray absorptiometry’ [1].

Our company, InBody Co. Ltd., manufacturer of the InBody device used for the above paper, has identified certain issues with the content and would appreciate your assistance in addressing them.

Questions have been raised about the comparison between ASMI (ASM) as measured by the InBody device and the corresponding measurements obtained through dual‐energy X‐ray absorptiometry (DEXA). In the paper, it was addressed that skeletal muscle mass (SMM) was directly used as appendicular skeletal muscle mass (ASM). However, SMM includes the muscles in the trunk, whereas the DEXA measurements considered only the muscle mass in the four limbs. In other words, ASM is appendicular skeletal muscle mass in the right arm, left arm, right leg and left leg, and SMM is trunk skeletal muscle mass added to this value.

Therefore, based on the definitions provided in the paper, there appears to be an inherent discrepancy between the ASM measurements obtained from InBody and those from DEXA, which could lead to an overestimation of ASM when utilizing InBody data for comparison. This fundamental difference in measurement methodologies has raised concerns about the accuracy of the comparisons made in the paper.

To explain with the result sheet that has been used in the paper (from inbodyusa.com), it reflects that the addition of the lean mass in all extremities adds up to 28.6 kg, but the SMM is 39.69 kg.

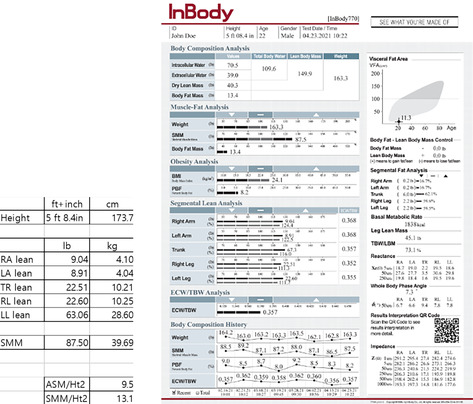

As shown above, the SMM/Ht^2^ and ASM/Ht^2^ values differ about 3.6, reflecting the fact that this example result sheet is of a muscular body type. For general people, we would expect from 2 to 3, also described in the paper.

Thus, as stated in the paper, ‘A significant overestimation of ASM, hence ASMI, was observed in measurements by the BIA compared with DXA (*p* < 0.005) (Table S1).’ would have been inevitable as the ASM derived from InBody would have included the trunk, and the ASM from DEXA would have not.

If it is determined that the definitions were inaccurately applied in the original paper, our team is interested in publishing a counter paper to provide a more accurate interpretation of the data. We kindly request information on the steps and guidelines for submitting such a counter paper, should that become necessary.

We appreciate your attention to this matter and look forward to your response. Your guidance and assistance in resolving this issue are highly valued, as we are committed to maintaining the integrity of scientific research in the field of Cachexia, Sarcopenia and Muscle.

Thank you for your time and consideration.

Sincerely,

Jade Kim
